# Multimodal assessment of exercise-induced fatigue: integrating cardiopulmonary, neuromuscular, and biomechanical profiling in high-intensity running

**DOI:** 10.3389/fspor.2026.1779542

**Published:** 2026-03-25

**Authors:** Yekai Wang, Ang Chen, Yichen Xu, Xindong Tao, Jihui Wang, Hui Li, Wei Ouyang

**Affiliations:** College of Physical Education and Health Sciences, Zhejiang Normal University, Jinhua, China

**Keywords:** electromyography, locomotor biomechanics, multimodal fatigue assessment, neuromuscular adaptation, plantar kinetics

## Abstract

This study aimed to establish a multimodal framework integrating cardiopulmonary exercise testing (CPET), surface electromyography (sEMG), and plantar kinetic assessment to characterize neuromuscular and mechanical fatigue responses during high-intensity treadmill exercise. Twenty healthy collegiate male athletes performed an incremental CPET followed by a supramaximal verification phase. Gas exchange, heart rate, and perceived exertion were continuously recorded. Bilateral sEMG activity from the rectus femoris, biceps femoris, tibialis anterior, and gastrocnemius lateralis was analyzed for integrated EMG (iEMG), root mean square (RMS), and median frequency (MF). Pre- and post-exercise plantar kinetics were obtained using in-shoe pressure sensors to assess contact area, mean pressure, and vertical ground reaction force (VGRF). Plantar kinetics showed increased midfoot contact area (+12.3%, *P* = 0.01) and pressure (+10.8%, *P* = 0.03), along with elevated left-foot regional VGRF (*P* = 0.04), indicating side-specific post-exercise load redistribution. Although nominal correlations were observed between neuromuscular activation and post-exercise plantar loading, these associations did not remain statistically significant after false discovery rate (FDR) correction and should therefore be interpreted as exploratory. Rather than establishing a quantitative diagnostic threshold for fatigue, integrating cardiopulmonary, neuromuscular, and plantar kinetic measures contributes to a multidimensional characterization of fatigue-related adaptations beyond metabolic indicators alone. This multimodal framework illustrates how neural drive decline and mechanical load redistribution may co-occur under acute fatigue conditions, providing a structured approach for comprehensive fatigue profiling in athletic populations.

## New & Noteworthy

This multimodal approach revealed synchronized cardiopulmonary stabilization, neuromuscular activation decline, and asymmetric midfoot load redistribution following exhaustive running. The observed associations among metabolic, neuromuscular, and biomechanical indices underscore fatigue as a coordinated multisystem phenomenon. Rather than establishing a quantitative diagnostic threshold, these findings contribute to a more comprehensive, multidimensional characterization of fatigue and provide a framework for integrating cardiopulmonary, neuromuscular, and biomechanical markers in athlete performance monitoring and injury-prevention strategies.

## 1 Introduction

Exercise-induced fatigue reflects a complex interaction between metabolic, neuromuscular, and biomechanical systems that progressively limits performance under maximal effort (1–3). Traditional cardiopulmonary exercise testing (CPET) quantifies physiological responses to graded workloads; however, the reliance on an oxygen uptake plateau as a definitive marker of maximal effort has been increasingly questioned (1, 2). Methodological variables such as ramp rate, sampling interval, and test design influence plateau manifestation, thereby reducing its diagnostic reliability (3, 4).

To address this limitation, supramaximal “verification” protocols have been developed to confirm maximal effort, though recent meta-analyses indicate that they add limited value in trained populations where reproducible oxygen uptake may already reflect physiological ceiling (3–5). Consequently, metabolic indices alone cannot fully capture peripheral and mechanical fatigue processes that contribute to exercise termination (6–8).

sEMG provides a sensitive index of neuromuscular function, allowing quantification of activation amplitude (RMS, iEMG) and spectral characteristics (median frequency) that reveal motor-unit recruitment decline, conduction slowing, and fiber-type transitions during fatigue (6–10). Concurrently, plantar kinetic analysis—including plantar pressure, contact area, vertical ground reaction force (VGRF), and foot progression angle (FPA)—offers biomechanical insight into load redistribution and altered gait strategies arising from local or systemic fatigue (11–16).

Integrating these modalities enables a comprehensive understanding of how central, peripheral, and mechanical systems adapt under fatigue. Accordingly, the present study aimed to combine CPET and a verification phase (VERT) with multi-channel sEMG and plantar kinetics to characterize pre- to post-exercise neuromuscular and mechanical fatigue in collegiate athletes. We hypothesized that fatigue would manifest as reduced muscle activation and increased midfoot loading, reflecting coordinated cardiopulmonary–neuromechanical adjustments to high-intensity exertion.

## 2 Methods

### 2.1 Ethical approval

Written informed consent was obtained from all participants after they were fully informed about the procedures, and any potential risks associated with the study. The study protocol was approved by the Zhejiang Normal University Human Research Ethics Committee (ZSRT2021071) in accordance with the Declaration of Helsinki. All participants provided written informed consent prior to participation.

### 2.2 Participants

Twenty healthy male athletes from the School of Physical Education, Zhejiang Normal University (mean age: 21.9 ± 1.7 years, mean height: 176.3 ± 4.1 cm, mean weight: 69.0 ± 6.8 kg), were recruited for this study. All were nonsmokers, regularly physically active, and free from cardiovascular, neurological, or musculoskeletal disorders. None had sustained lower-limb injuries within the previous six months or used performance-enhancing supplements. The detail basic information of participants was shown in Supplementary Table S1.

To ensure homogeneous physical fitness, participants completed standardized assessments of muscular endurance, strength, balance, flexibility, and mobility. On average, participants maintained single-leg balance for the full 60 s, performed 29.9 ± 5.9 sit-to-stand repetitions in 30 s, and completed 27.7 ± 3.8 right-arm curls with a 5 kg dumbbell in 30 s. Flexibility tests yielded a sit-and-reach distance of 12.8 ± 8.3 cm and a back-scratch distance of 4.5 ± 2.8 cm. Functional mobility and gait performance were stable, as indicated by a 7.1 ± 0.7 s time on the 2.45 m timed up-and-go and walking speeds of 1.5 ± 0.1 m·s^−^¹ (30 m test) and 1.2 ± 0.1 m·s^−^¹ (6 min walk). The detail fitness information of each participant was disclosed in Supplementary Table S2.

Body composition was evaluated using an InBody 570 analyzer (InBody USA, Cerritos, CA, USA), revealing a mean BMI of 22.1 ± 2.4 kg·m^−^², body fat percentage of 12.2 ± 4.9%, and basal metabolic rate of 7,532.2 ± 365.5 kJ. Participants with abnormal BMI values (≤18 or ≥25 kg·m^−^²) were excluded. All participants displayed normal foot morphology, verified via Arch Height Index (AHI = 0.275–0.365).

### 2.3 Study design

This study adopted a within-subject repeated-measures design to examine physiological, neuromuscular, and biomechanical adaptations to progressive and residual fatigue induced by maximal treadmill exercise. Each participant completed an incremental cardiopulmonary exercise test (CPET) followed, after 10 min of recovery, by a supramaximal verification phase (VERT). sEMG signals were recorded bilaterally from the rectus femoris, biceps femoris, tibialis anterior, and gastrocnemius lateralis throughout both CPET and VERT to capture real-time neuromuscular activation patterns.

These muscles were selected because they represent major functional contributors to sagittal-plane locomotion during treadmill running. The rectus femoris and biceps femoris form a primary agonist–antagonist pair at the knee joint, playing essential roles in knee extension and flexion during propulsion and deceleration phases. The tibialis anterior and gastrocnemius lateralis were included due to their critical involvement in ankle dorsiflexion and plantarflexion, respectively, contributing to foot stabilization, shock absorption, and push-off power generation. Together, these muscle groups provide a representative profile of proximal and distal lower-limb neuromuscular function and are known to be sensitive to fatigue during high-intensity running.

The plantar kinetic data were collected immediately before (Pre) and after (Post) the entire CPET–VERT protocol to detect fatigue-induced alterations in foot loading and symmetry. These pre-to-post comparisons, together with the within-test sEMG changes, enable characterization of fatigue as a graded process, transitioning from cardiopulmonary strain to neuromechanical compensation.

**Figure 1:**
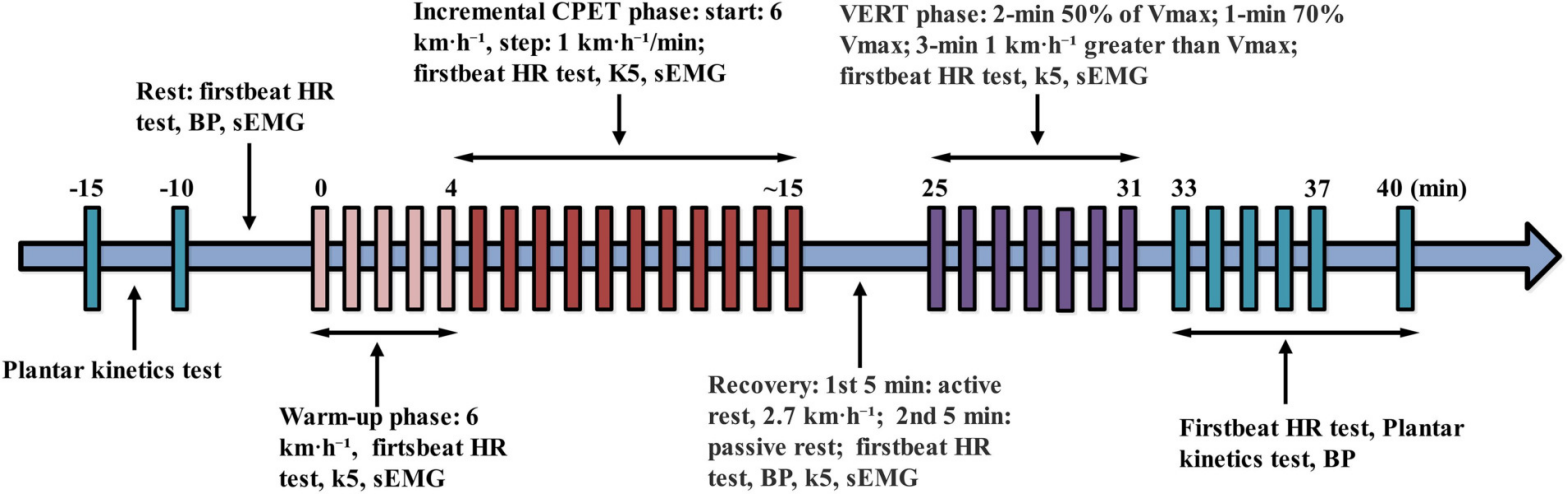
The experimental timeline. BP, blood pressure test; CPET, cardiopulmonary exercise testing; HR, heart rate; K5, COSMED K5 portable metabolic analyzer; VERT, verification Phase Test; sEMG, surface electromyography.

All tests were conducted in the Exercise Physiology Laboratory at Zhejiang Normal University under controlled conditions (23 ± 1 °C; 50%–55% humidity) and at a consistent time of day to minimize circadian effects. This within-condition comparison enabled evaluation of acute fatigue effects (Pre vs. Post) alongside phase-specific differences (CPET vs. VERT) in the same individuals. The multimodal framework thus provided an integrative profile of cardiopulmonary stabilization, neuromuscular decline, and plantar load redistribution under maximal exertion, providing an integrated profile of fatigue development during maximal treadmill running. The timeline of the study design is shown in Figure 1.

#### 2.3.1 Warm-up and cardiopulmonary exercise testing (CPET)

Participants visited and conducted CPET/VERT test in human performance laboratory at same time in one week, and the laboratory condition was kept constant, with temperature of 22 ± 2 °C and humidity of 35% ± 5%. Participants first rest in chair for 10 min, then completed a 5 min warm-up on a treadmill at a constant speed of 6 km·h^−^¹. Following the warm-up, participants underwent an incremental CPET using a treadmill (Pulsar, h/p/cosmos, Germany). The protocol consisted of a stepwise increase in treadmill speed by 1 km·h^−^¹ every minute, starting from 6 km·h^−^¹, until participants reached their maximal effort. The protocol of rest warm-up and increment phases took approximately 15–20 min depending on each participant's ability to catch up and maintain the running speed. Subsequently, participants were allowed for 10 min recovery including 5 min active rest (walk on treadmill at 2.7 km·h^−^¹), followed by 5 min passive rest (sit in a chair for 5 min). The target for CPET was to reach maximal oxygen uptake (VO_2_max), which was determined by the highest oxygen consumption (VO_2_) value achieved, or a plateau in VO_2_ with a corresponding increase in VE and VCO_2_ production.

#### 2.3.2 Verification phase test (VERT)

After completing the CPET, participants were allowed a 10 min recovery (5 min active rest and 5 min passive rest). They then underwent the VERT, which aimed to confirm the VO_2_max results obtained during the CPET. VERT protocol was adapted according to a previous report (17). In the VERT, participants began by running at 50% of the maximal speed achieved during CPET for 2 min, followed by a 70% maximal speed run for 1 min. Finally, participants ran at a speed 1 km·h^−^¹ greater than the maximal speed achieved during CPET for 3 min. The VERT protocol took approximately 4.1–5.2 min depending on each participant's ability to catch up and maintain the running speed. The VERT was designed to ensure that the athlete was working at the maximal capacity, and the VO_2_max was re-validated based on the peak VO_2_ during this test. After VERT, the Borg 6–20 rating of perceived exertion (RPE) scale was assessed with each participant.

#### 2.3.3 Cardiopulmonary data collection and analysis

During the 10 min rest prior to warm-up phase, the participants were fitted with alcohol disinfected breathing mask. The gas exchange was measured continuously by using a COSMED K5 portable metabolic analyzer (firmware 2.1, COSMED, Italy) throughout the rest, warm-up, incremental CPET and recovery (passive rest) phases. The K5 system consisted with an oxygen galvanic fuel cell (technical accuracy, ±0.02%; response time, 120 ms), digital infrared carbon dioxide sensor (±0.01%, 100 ms), bidirectional optoelectronic turbine flow sensor (±2.00%), a 2 mL dynamic micro mixing chamber (DMC), and transmits data through Bluetooth approach. The DMC mode provides superior validity in assessing VO_2_ consumption at high metabolic rates during high intensity activity (18). The K5 analyzer was calibrated according to manufacturer's instruction prior to each measurement. Gas exchange parameters, including VO_2_, VCO_2_, VE, and the respiratory exchange ratio (RQ), were continuously monitored using DMC mode in the K5 analyzer. VO_2_, VCO_2_, VE, and RQ values were calculated in 10 s interval based on 30 s rolling average of mixing chamber gases throughout CPET and VERT testing processes. The final of 60 s rates during each phase of aforementioned CPET and VERT protocols were used for analysis.

#### 2.3.4 Cardiopulmonary identifications

A VO2-pl was defined as a change in oxygen uptake (*Δ*VO₂) of less than 2.1 mL·min^−^¹·kg^−^¹ between the average VO₂ values of the last and penultimate 30 s periods during the incremental treadmill phase (1, 19). However, several factors such as exercise protocol, subject characteristics, training status, and breath-by-breath averaging strategy may affect the efficacy of identification of VO2-pl, therefore the VO2-pl may reflect a calculation, raising the possibility that some detected plateaus represent calculation artifacts rather than true physiological phenomena (1, 20). Because not all participants exhibit a clear plateau even at maximal effort, secondary criteria were also applied to corroborate VO₂max attainment. These included: (i) the last two measured respiratory exchange ratios (RER ≥ 1.10 during CPET, ≥1.00 during VERT), (ii) peak heart rate within 10 beats·min^−^¹ of the age-predicted maximum, and (iii) Borg rating of perceived exertion (RPE ≥ 18). In the absence of a plateau, fulfillment of at least two secondary criteria was required for VO_2_max confirmation (1, 5).

In this study, we focus more on the changes in maximum oxygen uptake and its correlation with other physiological indicators, and VO_2_max was defined as the highest within last 60 s during CPET or VERT (5). The K5 metabolic analyzer was set to compute gas exchange variables using short averaging intervals (10–30 s), as recommended for improving sensitivity to plateau detection (20). Finally, the VERT served as an external validation. Attainment of similar or higher VO_2_ values during VERT compared with CPET was considered confirmatory for VO_2_max determination (1, 17).

#### 2.3.5 Heart rate (HR) and blood pressure (BP) measurements

Genetic differences are known to influence physical parameters, such as height, weight, and body mass index, and thereby maximal heart rate (HRmax) values (21). Therefore, we applied a recently validated formula for young Asian men (aged 15–24 years): HRmax = 214 −0.8 × age (22). Actual HR was continuously recorded using a Firstbeat wireless chest-belt monitor (Transmission protocol, 915/868 MHz and BTLE 4.0; Firstbeat Technologies Ltd., Jyväskylä, Finland). Maximum HR during CPET and VERT phases were extracted for analysis. These HR metrics were used to compare maximal cardiac responses between CPET and VERT, evaluate proximity to the age-predicted HRmax, and confirm physiological consistency across test phases.

Right brachial artery blood pressure (BP) was measured twice using an automated sphygmomanometer (HEM-7121, Omron Healthcare, Lake Forest, IL, USA) and averaged at 10 min before and after the experimental protocol in a supine position. All measurements were performed under standardized laboratory conditions. The detail HR and BP metrics of each participant at pre-exe and post-exe phases were shown in Supplementary Table S3.

#### 2.3.6 Electromyography (EMG) recording and data processing

sEMG was used to monitor neuromuscular activation during the final 2 min of CPET and VERT. A BTS FREEEMG 300 wireless system (BTS Bioengineering, Milan, Italy) recorded activity from the rectus femoris (RF), biceps femoris (BF), tibialis anterior (TA), and gastrocnemius lateralis (GL) bilaterally. The wireless miniaturized probes were placed on the muscle belly after skin preparation (cleaning and shaving) and secured with medical gauze. EMG signals were sampled at 1,000 Hz (S/N = 96 dB, common-mode rejection ratio >110 dB at 50–60 Hz, sensitivity = 1 μV, accuracy ±2%). Signals were band-pass filtered (20–450 Hz) and processed with BTS EMG-Analyzer software.

The following parameters were extracted from the last 60 s of CPET and VERT: integrated EMG (iEMG) and RMS amplitude (primary indices for motor unit recruitment and metabolic threshold detection), and mean frequency (a supportive index of fatigue-related decline in conduction velocity). Additionally, a relative activation ratio was computed as the ratio of GL RMS to TA RMS (GL/TA RMS). This ratio was used as an index of relative activation ratio (RAR) between the plantarflexor (gastrocnemius lateralis) and dorsiflexor (tibialis anterior) muscle groups during treadmill running. Given the complementary roles of these muscles in ankle stabilization and propulsion during gait, shifts in their relative activation may reflect fatigue-related adjustments in distal muscle recruitment patterns.

sEMG signals were not normalized to maximal voluntary contraction (MVC) or peak dynamic reference values. Instead, absolute iEMG, RMS, and median frequency values were analyzed within subjects across exercise phases (CPET vs. VERT). This approach was adopted because the study employed a within-session repeated-measures design in which electrode placement was maintained without repositioning throughout the entire testing session, and recording conditions were standardized to minimize impedance variability. Given this design, comparisons were restricted to within-subject phase differences rather than between-subject activation magnitude comparisons.

#### 2.3.7 Plantar kinetics testing and analysis

Fifteen minutes before the participants performed CPET and immediately after completing the VERT, plantar kinetics were assessed using a Footscan 1.5 m pressure mat (RSscan International, Olen, Belgium, sampling rate 300–400 Hz; number of sensors: 12,288/1.5 m, sensor size: 5 × 7.6 mm, 4 sensors/cm², pressure range: 0.7–155 N/cm², accuracy: 3.3%). This methodological choice was based on the fact that the Footscan system is validated primarily for walking gait analysis rather than high-speed running. Walking trials allowed standardized stance-phase segmentation, reliable regional plantar pressure acquisition, and controlled comparison of pre- and post-fatigue mechanical adaptations. Accordingly, plantar kinetic outcomes reflect post-exercise walking gait mechanics rather than in-run kinetics. The pressure mat was placed in the center of a 5-meter-long walkway. Participants began walking with the right foot first, while the left foot stepped onto the pressure mat. To avoid restricting lower limb movements, participants were instructed to wear loose, comfortable clothing and perform a 3 min warm-up walking on the pressure mat and walkway before data collection (23).

During the test, participants were instructed to walk at a comfortable, natural speed across the 5-meter walkway, completing the walk at the far end of the mat. To enhance the reliability of the data, each participant completed three trials, and the average values were used for subsequent analysis (24). The parameters recorded included foot contact area (cm²), foot pressure (N/cm²), Force Time Integral (FTI), VGRF, and Foot Progression Angle (FPA). In the present study, VGRF values represent regional peak vertical forces detected within specific plantar regions by the Footscan pressure mat system, rather than whole-body ground reaction forces measured by force platforms. These values were not normalized to body weight. The test was considered valid if the following criteria were met: (i) at least two complete footprints were present on the pressure mat, (ii) a clear heel strike pattern was observed during walking, and (iii) no significant posture adjustments were made during walking on the mat (25).

The Footscan system software was used to assess plantar pressure distribution across ten-foot regions, including the hallux (T1), toes 2–5 (T2–T5), the first metatarsal (M1), second metatarsal (M2), third metatarsal (M3), fourth metatarsal (M4), fifth metatarsal (M5), midfoot (MF), medial heel (MH), and lateral heel (LH) (26). The force time integral (FTI) was defined as the impulse (N.s) generated by the force applied over time in a specific region of the foot. It has been widely used to quantify cumulative loading in the foot, which can contribute to tissue damage and injury risk (27). The FPA was calculated as the angle between the long axis of the foot, defined from the heel to the second metatarsal, and the line of progression during walking. Positive FPA values indicate internal rotation, while negative values indicate external rotation, both of which can indicate fatigue and compensatory gait adjustments (27). Foot rotation during walking increases the load on structures such as bones, ligaments, and muscles, thereby raising the risk of injury (27, 28). Therefore, besides assessing contact area and pressure, this study also evaluated changes in FTI, VGRF, and FPA to assess the effects of fatigue on foot biomechanics. During the testing phase, all participants performed walking trials with the same timing, from heel strike to the toe-off phase. The first and second VGRF peaks were collected during these phases for further analysis. The foot contact area, pressure, FTI, FPA, and the HR results were independently assessed by three blinded evaluators.

### 2.4 Statistical analysis

Sample size (*n* = 20) was determined by convenience from the available collegiate athlete cohort. A *post hoc* power analysis performed in G*Power (version 3.1.9.7) indicated that, for paired comparisons assuming a moderate effect size (Cohen's *d* = 0.5), *α* = 0.05 (two-tailed), and *n* = 20, the statistical power (1–β) was 0.75, considered acceptable for detecting medium effects in the primary outcomes (V̇O₂max, sEMG, and plantar-kinetic variables).

All data are expressed as mean ± SD. Normality of distributions was verified with the Shapiro–Wilk test and visual inspection of *Q*–*Q* plots. For non-normally distributed variables, equivalent nonparametric tests were applied. Cardiopulmonary variables (VO_2_, RER, HR) across test phases (rest, warm-up, CPET, recovery, VERT) were analyzed using two-way repeated-measures ANOVA (factors: phase × time), with Greenhouse–Geisser correction for sphericity violations. Repeated-measures ANOVA *post hoc* comparisons were adjusted using Bonferroni correction. Agreement between VO_2_max values from CPET and VERT was further examined using Bland–Altman analysis and Pearson correlation. Simple linear regression analyses were used to characterize the slopes for VO_2_-time curves during CPET and VERT. sEMG parameters, such as iEMG, RMS, median frequency, and the relative activation ratio (RAR; GL RMS/TA RMS), were analyzed with two-way repeated-measures ANOVA (factors: muscle × phase), followed by Bonferroni-adjusted *post hoc* tests. A within-subject design was adopted to compare plantar-kinetic variables (contact area, mean plantar pressure, FTI, VGRF, FPA) using two-way repeated-measures ANOVA (region × time), and paired *t*-tests where significant interactions occurred.

Correlational analyses were performed using Pearson's coefficients. Associations were examined between VO₂max and (1) sEMG indices (iEMG, RMS, median frequency) and (2) plantar kinetic variables (contact area, pressure, VGRF). To control for multiple comparisons across correlational analyses (m = 7 tests), the Benjamini–Hochberg (BH) false discovery rate (FDR) procedure was applied with a desired FDR (Q) of 0.05. Effect sizes were calculated from the observed data and expressed as Cohen's *d* for paired and repeated-measures comparisons, interpreted as small (0.2), medium (0.5), or large (≥0.8). Statistical significance was set at *P* < 0.05. All analyses were conducted using GraphPad Prism 9 (GraphPad Software, La Jolla, CA, USA).

## 3 Results

### 3.1 Cardiopulmonary responses

A distinct VO₂-pl was identified in 17 participants during the incremental CPET and in 18 participants during the subsequent VERT, based on the criterion of ΔVO₂ < 2.1 mL·min^−^¹·kg^−^¹ between the last and penultimate 30 s averaging intervals. To further confirm the attainment of maximal effort, secondary VO_2_max verification criteria were applied. During CPET, all participants achieved a respiratory exchange ratio (RER) ≥ 1.10, whereas during VERT, 16 participants exhibited RER values ≥1.00. Peak heart rate (HR) values within 10 beats·min^−^¹ of the age-predicted maximum were observed in 13 participants during CPET and 15 participants during VERT. Moreover, ratings of perceived exertion (RPE) were ≥18 (Supplementary Table S4) in all participants across both test phases, corroborating maximal perceived effort.

**Figure 2:**
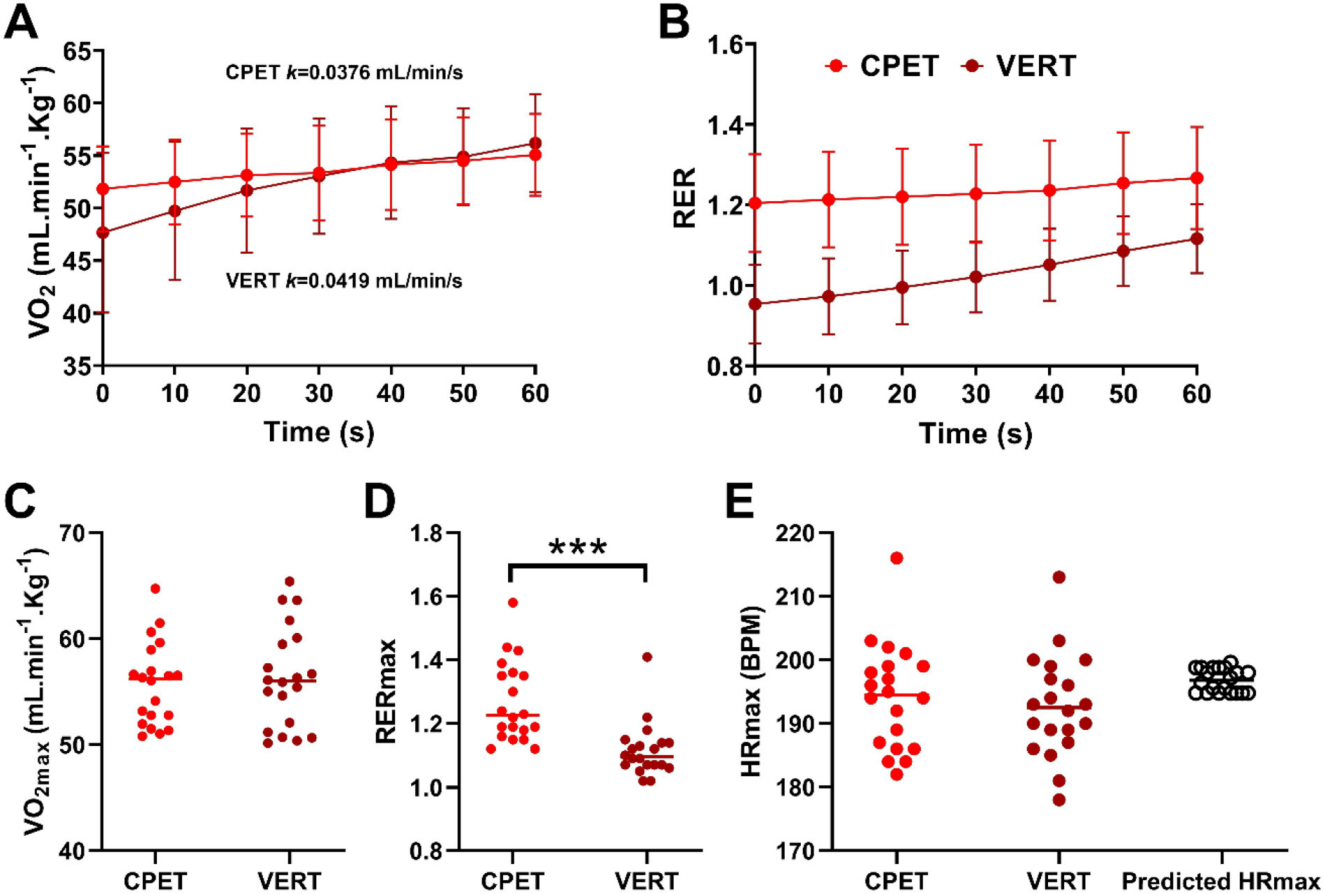
Cardiopulmonary responses during the incremental CPET and VERT phase. **(A)** VO₂ values during the final 60 s of CPET and VERT. **(B)** Respiratory exchange ratio (RER) values during the final 60 s of CPET and VERT. **(C)** Comparison of relative VO₂max values between CPET and VERT. **(D)** Comparison of RERmax values between CPET and VERT. **(E)** Comparison of HRmax values between CPET and VERT. Data are presented as mean ± SD (*n* = 20). ***P < 0.001 vs. CPET (paired *t*-test). CPET, cardiopulmonary exercise test; RER, respiratory exchange ratio; VERT, verification phase test.

As shown in Figures 2A,B, linear regression of VO_2_–time data during the final 60 s of both phases demonstrated negligible upward trends, with slopes (k) of 0.0376 mL·min^−^¹·s^−^¹ (R² = 0.021) for CPET and 0.0419 mL·min^−^¹·s^−^¹ (R² = 0.035) for VERT, consistent with physiological stabilization. The marginally steeper slope during VERT was not statistically significant, indicating reproducibility of the plateau response. Moreover, VO_2_ and RER were significantly correlated in final 60 s during both CPET (*r* = 0.535, *P* = 0.015) and VERT (*r* = 0.677, *P* = 0.001), demonstrating synchronized metabolic and ventilatory adjustments at maximal exertion.

The mean relative VO_2_max was 55.7 ± 3.82 mL·kg^−^¹·min^−^¹ during CPET (VO_2_max-CPET) and 56.3 ± 4.64 mL·kg^−^¹·min^−^¹ during VERT (VO₂max-VERT), with no significant difference between phases (*P* = 0.40, paired *t*-test, *d* = 0.14; Figure 2C). Pearson correlation analysis revealed a strong positive association between the two measures (*r* = 0.73, *P* = 0.0003; BH adjusted *P* = 0.0021, Supplementary Table S5), accounting for approximately 53% of shared variance (R² = 0.53) and indicating good consistency between protocols. Bland–Altman analysis further indicated minimal systematic bias (mean bias = –0.63 ± 3.28 mL·kg^−^¹·min^−^¹), confirming high agreement and reproducibility between CPET- and VERT-derived VO_2_max measurements.

By contrast, mean RERmax values were significantly higher during CPET (1.27 ± 0.12) compared with VERT (1.12 ± 0.08; *P* = 0.001, *d* = 1.45; Figure 2D), reflecting greater degree of hyperventilatory drive during incremental loading. Maximal HR was slightly higher during CPET (194.2 ± 8.08 beats·min^−^¹) compared with VERT (192.8 ± 7.84 beats·min^−^¹; Figure 2E), though both values were within ∼2–4 beats·min^−^¹ of the age-predicted HRmax (196.8 ± 1.78 beats·min^−^¹). One-way ANOVA revealed no significant differences among these HRmax estimates (*P* > 0.05), demonstrating physiological consistency across tests.

Interestingly, correlation analyses revealed that the relationship between VO_2_max and HR was stronger during VERT than CPET. During CPET, VO_2_max was weakly and non-significantly related to HRmax (*r* = –0.273, *P* = 0.244 for VO_2_max–HRmax-CPET; *r* = –0.276, *P* = 0.239 for VO_2_max–HRmax-VERT). In contrast, during VERT, these relationships became more pronounced (*r* = –0.450, *P* = 0.046 for VO_2_max-VERT–HRmax-CPET; *r* = –0.399, *P* = 0.081 for VO_2_max-VERT–HRmax-VERT), suggesting that the verification phase elicited a tighter coupling between oxygen uptake and cardiac response under stabilized maximal conditions.

Collectively, these findings confirm strong concordance between CPET and VERT for determining maximal oxygen uptake, validating the verification phase as a reliable and physiologically consistent approach for confirming true VO_2_max attainment.

### 3.2 Neuromuscular activation and fatigue dynamics

**Figure 3:**
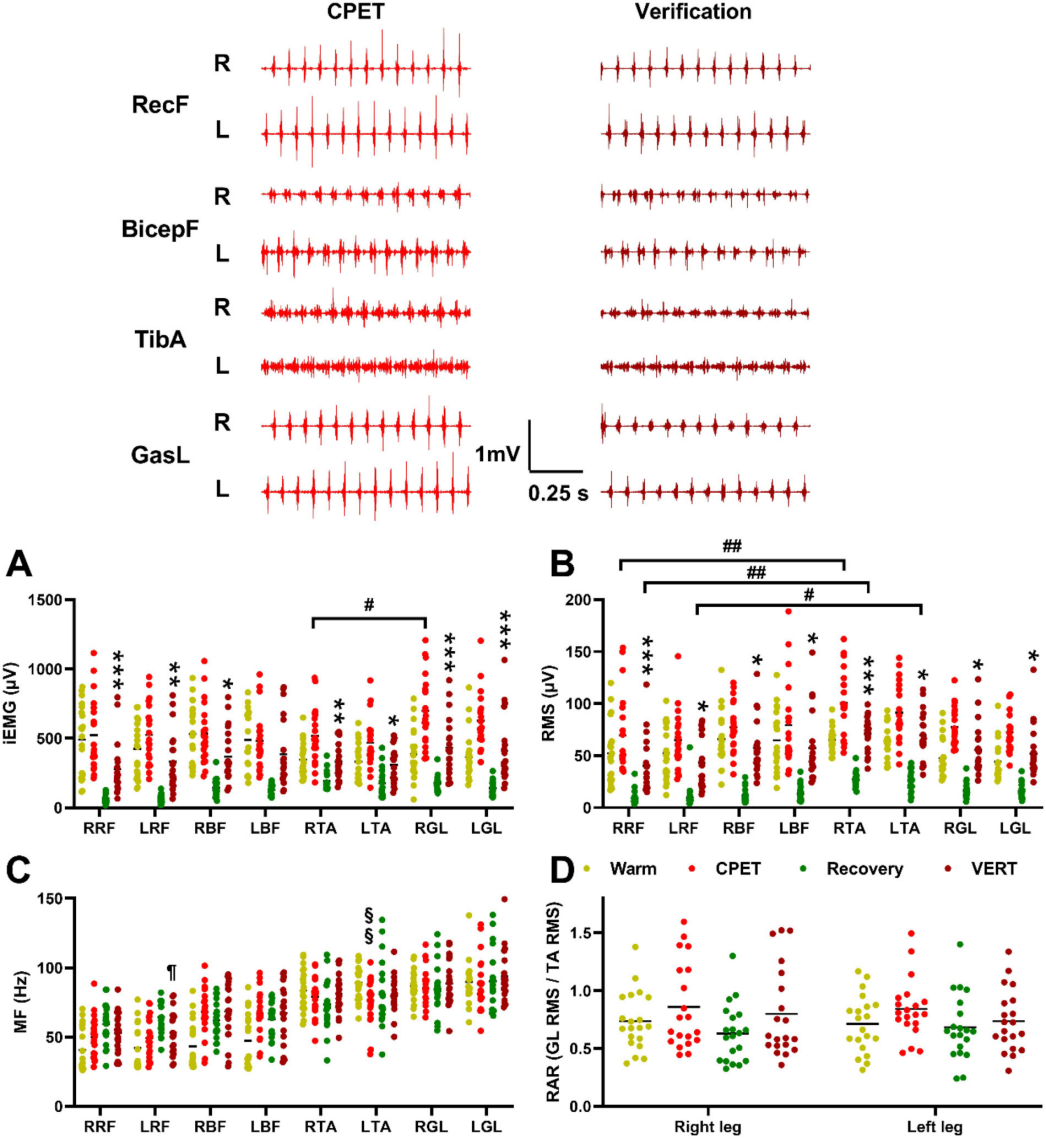
sEMG parameters across warm-up, incremental CPET, recovery, and VERT phase. The top panel displays representative raw sEMG recordings from thigh (RF and BF) and calf (TA and GL) muscles. **(A)** iEMG values during warm-up, CPET, recovery, and VERT. **(B)** RMS values during warm-up, CPET, recovery, and VERT. **(C)** Median frequency values during warm-up, CPET, recovery, and VERT. **(D)** Coordination ratio of GL RMS vs. TA RMS. Data is presented as mean ± SD (*n* = 18). *P < 0.05, **P < 0.01, ***P < 0.001 for comparisons between CPET and VERT phases; ^§§^*P* < 0.01 for comparison between warm and CPET phases; ^¶^*P* < 0.05 for comparison between recovery and VERT phases; ^#^*P* < 0.05, ^##^*P* < 0.01 vs. left or right thigh and calf muscles (two-way ordinary or repeated-measures ANOVA with Bonferroni *post hoc* test). RF, rectus femoris; BF, biceps femoris; TA, tibialis anterior; GL, gastrocnemius lateralis; iEMG, integrated electromyography; RMS, root mean square; RAR, relative activation ratio (RAR; GL RMS/TA RMS); CPET, cardiopulmonary exercise test; VERT, verification phase test.

sEMG signals recorded throughout the warm-up, CPET, recovery, and VERT phases were analyzed to evaluate neuromuscular activation and fatigue responses in major thigh and calf muscles (Figure 3). The primary parameters—iEMG and RMS—reflect the magnitude and temporal characteristics of muscle recruitment. While iEMG emphasizes the cumulative activation over time, RMS highlights the instantaneous amplitude and contractile intensity of motor unit activity.

Two-way repeated-measures ANOVA revealed a significant main effect of phase (warm-up, CPET, recovery, VERT) on both iEMG and RMS across all muscle groups (*P* < 0.001). *post hoc* pairwise comparisons (Bonferroni correction) indicated that iEMG values were significantly higher during CPET than during VERT in the right and left rectus femoris (RRF, LRF; both *P* < 0.001), right biceps femoris (RBF; *P* < 0.05), right and left tibialis anterior (RTA, *P* < 0.01; LTA, *P* < 0.05), and both gastrocnemius lateralis muscles (RGL, LGL; both *P* < 0.001, *d* = 1.14; Figure 3A). Similarly, RMS values were significantly elevated during CPET relative to VERT for RRF (*P* < 0.001), LRF (*P* < 0.05), RBF and LBF (both *P* < 0.05), RTA (*P* < 0.001, *d* = 1.18), LTA (*P* < 0.05), and both RGL and LGL (both *P* < 0.05; Figure 3B). These findings demonstrate a global reduction in cumulative and instantaneous muscle activation during the VERT phase, indicating partial neuromuscular fatigue following the maximal incremental load.

Peak iEMG values were observed in the gastrocnemius lateralis during CPET (RGL: 699.26 ± 248.88 μV; LGL: 626.29 ± 248.88 μV) and decreased during VERT (RGL: 440.46 ± 204.21 μV; LGL: 421.76 ± 226.57 μV). In contrast, the tibialis anterior exhibited the greatest RMS amplitudes in both phases (CPET: RTA, 100.53 ± 31.77 μV; LTA, 91.37 ± 27.91 μV; VERT: RTA, 71.32 ± 15.86 μV; LTA, 69.77 ± 21.98 μV). These patterns suggest that the gastrocnemius lateralis contributed most to total workload, while the tibialis anterior was dominant in dynamic amplitude and postural stabilization during high-intensity running.

Pearson correlation analysis revealed that VO_2_max was significantly and positively associated with iEMG in the left tibialis anterior during CPET (*r* = 0.470, *P* = 0.037) and showed a positive trend with iEMG in the right biceps femoris during VERT (*r* = 0.430, *P* = 0.058). During VERT, VO_2_max also correlated positively with RMS in the right biceps femoris (*r* = 0.468, *P* = 0.038) and negatively with median frequency in the left tibialis anterior (*r* = −0.447, *P* = 0.048). These results suggest that enhanced recruitment intensity in the biceps femoris and tibialis anterior contributes to higher oxygen uptake during maximal and supramaximal exertion, while median frequency declines mirror fatigue-related reductions in conduction velocity. Thus, iEMG and RMS appear to be the most sensitive indicators of VO_2_max attainment and exercise intensity, whereas median frequency provides supportive evidence of fatigue progression.

Pearson correlation analyses revealed several nominal associations between VO₂max and selected neuromuscular parameters. VO_2_max was positively associated with iEMG in the left tibialis anterior during CPET (*r* = 0.470, *P* = 0.037) and with RMS in the right biceps femoris during VERT (*r* = 0.468, *P* = 0.038). A negative association was observed between VO_2_max and median frequency in the left tibialis anterior during VERT (*r* = −0.447, *P* = 0.048), while a positive trend was noted for iEMG in the right biceps femoris during VERT (*r* = 0.430, *P* = 0.058). These patterns suggest that individuals with higher aerobic capacity may exhibit greater neuromuscular activation intensity during maximal and supramaximal exertion, alongside frequency-domain changes consistent with fatigue-related alterations in motor unit conduction velocity. The convergence of amplitude-based indices (iEMG and RMS) with VO_2_max may reflect enhanced motor unit recruitment or firing synchronization during high-intensity efforts.

However, after applying the BH-FDR correction (*Q* = 0.05), none of these associations remained statistically significant (all adjusted *P* ≥ 0.056, Supplementary Table 5S). Given the moderate effect sizes (*r* ≈ 0.43–0.47) and the relatively small sample size (*n* = 20), statistical power was limited following correction for multiple comparisons. Therefore, these findings should be interpreted as preliminary indications of a potential coupling between aerobic capacity and neuromuscular activation strategies rather than definitive evidence. Further studies with larger cohorts and pre-specified primary outcomes are warranted to clarify whether these neuromuscular patterns represent robust physiological adaptations associated with superior VO_2_max attainment.

### 3.3 Fatigue trends and neuromuscular coordination

Median frequency, reflecting average motor unit firing rate and conduction velocity, decreased progressively with exercise intensity and fatigue. The largest median frequency decline occurred in the tibialis anterior between the warm-up and CPET (RTA: 83.62 ± 14.76–79.02 ± 13.02 Hz; LTA: 89.00 ± 12.11–76.27 ± 16.04 Hz, *P* < 0.01; Figure 3C), while the steepest reduction from recovery to VERT was found in the rectus femoris (RRF: 59.26 ± 14.20–52.78 ± 13.99 Hz; LRF: 64.42 ± 11.37–53.18 ± 15.24 Hz, *P* < 0.05; Figure 3C). These reductions confirm that the tibialis anterior and rectus femoris were more susceptible to peripheral fatigue during incremental and verification running, respectively.

Although the GL/TA RMS ratio did not reach statistical significance, both right and left values exhibited a consistent increasing trend from the warm-up to CPET and VERT. This upward tendency suggests a relative increase in plantarflexor activation compared with dorsiflexor activation as fatigue progressed, indicating altered distal muscle recruitment distribution during high-intensity treadmill running.

### 3.4 Plantar kinetic alterations following the complete exercise protocol

Compared with the Pre-test condition, the Post-test showed significant increases in midfoot contact area (+12.3%, *P* = 0.01, *d* = 0.74) and mean pressure (+10.8%, *P* = 0.03, *d* = 0.68), along with a rise in left-foot peak VGRF (*P* = 0.04). These results confirm that the CPET–VERT sequence induced measurable mechanical fatigue reflected in plantar load redistribution.

**Figure 4:**
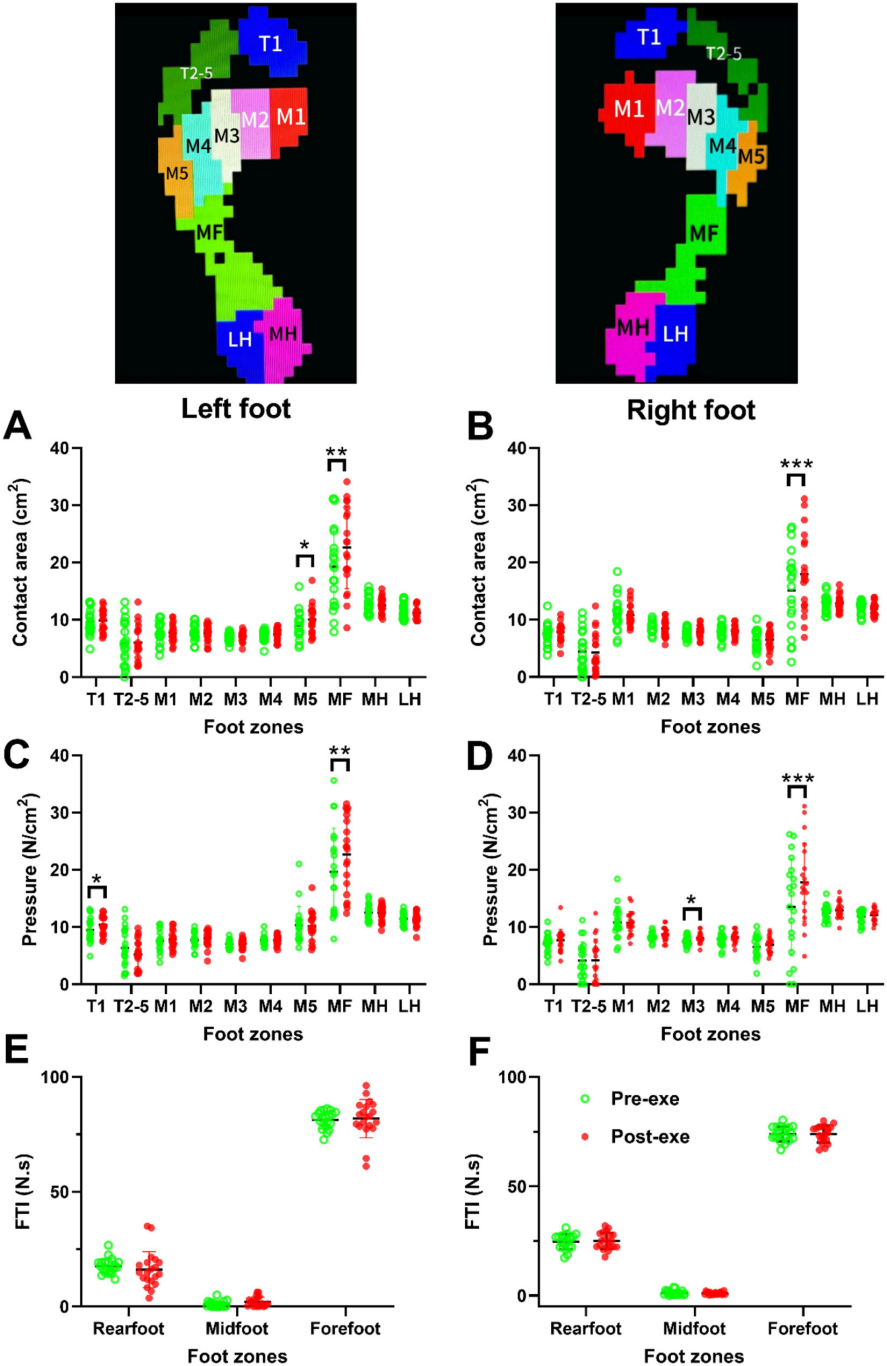
Plantar kinetic parameters pre- and post-the entire exercise protocol. **(A)** Representative images of foot subdivision regions in the left and right feet. Subdivisions include T1, T2-5, M1, M2, M3, M4, M5, MF, MH, and LH. **(B)** Contact area values pre- and post-CPET/VERT exercise. **(C)** Plantar pressure values pre- and post-CPET/VERT exercise. **(D)** FTI values in forefoot, midfoot, and rearfoot pre- and post-CPET/VERT exercise. Data are presented as mean ± SD (*n* = 20). *P < 0.05 by paired *t*-test. CPET, cardiopulmonary exercise test; FTI, force-time integral; LH, lateral heel; M1, first metatarsal; M2, second metatarsal; M3, third metatarsal; M4, fourth metatarsal; M5, fifth metatarsal; MF, midfoot; MH, medial heel; T1, hallux; T2–5, toes 2–5; VERT, verification test.

In the left foot, the midfoot (MF) region exhibited a significant increase in contact area from 19.25 ± 6.93 cm² to 22.61 ± 6.98 cm² (*P* < 0.05, *d* = 0.48), and the fifth metatarsal (M5) region also showed an increase from 9.00 ± 2.34 cm² to 10.08 ± 2.51 cm² (*P* < 0.05; Figure 4A). Comparable alterations were observed in the right foot, where the MF region increased from 15.15 ± 6.95 cm² to 17.97 ± 6.72 cm² (*P* < 0.001; Figure 4B). These changes suggest enhanced midfoot engagement and lateral forefoot loading during post-fatigue gait.

Analogous trends were evident in plantar pressure distribution. A significant region × time interaction effect was found (*P* < 0.001). In the left foot, the MF region exhibited a marked increase in mean plantar pressure from 19.69 ± 7.41 N/cm² to 22.68 ± 6.37 N/cm² (*P* < 0.001, *d* = 0.43; Figure 4C). Subsequent paired *t*-tests further identified a significant increase in pressure under the hallux (T1) region (from 9.47 ± 2.08 N/cm² to 10.40 ± 1.54 N/cm², *d* = 0.51; *P* < 0.05), accompanied by a significant decrease under the lesser toes (T2–5) region (from 6.35 ± 3.38 N/cm² to 5.21 ± 2.47 N/cm²; *P* < 0.05, *d* = 0.39; Figure 4C).

In the right foot, plantar pressure significantly increased in the MF region (from 13.51 ± 8.24 N/cm² to 17.85 ± 6.66 N/cm²; *P* < 0.001, *d* = 0.58; Figure 4D) and in the M3 region (from 7.58 ± 0.82 N/cm² to 8.10 ± 0.87 N/cm², *d* = 0.61; *P* < 0.05). FTI analyses further supported these findings. Post-exercise, the FTI of midfoot region demonstrated an upward trend in the left foot (1.03 ± 1.26–2.01 ± 2.05 N·s), while the FTI of midfoot region in right foot subtly changed from (1.25 ± 1.03–1.06 ± 0.58 N·s, Figures 4E,F). The FTI values of forefoot and rearfoot in left (forefoot: 81.36 ± 3.65–81.88 ± 8.09 N·s; rearfoot: 17.61 ± 3.31–16.11 ± 7.67 N·s) and right (forefoot: 73.99 ± 3.26–73.91 ± 3.77 N·s; rearfoot: 24.74 ± 3.40–25.03 ± 3.72 N·s, Figures 4E,F) foot remain stable.

Collectively, these patterns reflect greater impact loading in midfoot region, typically in left foot, following exhaustive treadmill exercise. Correlation analyses demonstrated that post-exercise midfoot contact area (*r* = –0.470, *P* = 0.047) and pressure (*r* = –0.477, *P* = 0.034) were significantly and negatively associated with aerobic capacity, suggesting that participants with superior endurance exhibited reduced midfoot loading and pressure, potentially reflecting more efficient energy transfer and better fatigue resistance during high-intensity locomotion.

Collectively, these patterns indicate fatigue-related alterations in midfoot loading during post-exercise gait. Pearson correlation analyses demonstrated nominal negative associations between VO₂max (VERT) and post-exercise right midfoot contact area (*r* = –0.470, *P* = 0.047) as well as midfoot pressure (*r* = –0.477, *P* = 0.034), suggesting that individuals with higher aerobic capacity tended to exhibit reduced midfoot loading following exhaustive treadmill exercise. However, after applying the BH-FDR correction (*Q* = 0.05), these associations did not remain statistically significant (adjusted *P* ≥ 0.056, Supplementary Table 5S). Therefore, these findings should be interpreted as exploratory trends rather than definitive evidence of biomechanical efficiency differences related to aerobic capacity.

Although the observed effect sizes were moderate, the relatively small sample size (*n* = 20) and adjustment for multiple comparisons likely reduced statistical power. Nevertheless, the consistent directionality of the associations may tentatively suggest that individuals with superior endurance capacity adopt locomotor strategies characterized by reduced midfoot loading under fatigued conditions. Confirmation of this potential relationship requires larger-scale studies with predefined primary biomechanical endpoints.

### 3.5 VGRF dynamics

**Figure 5:**
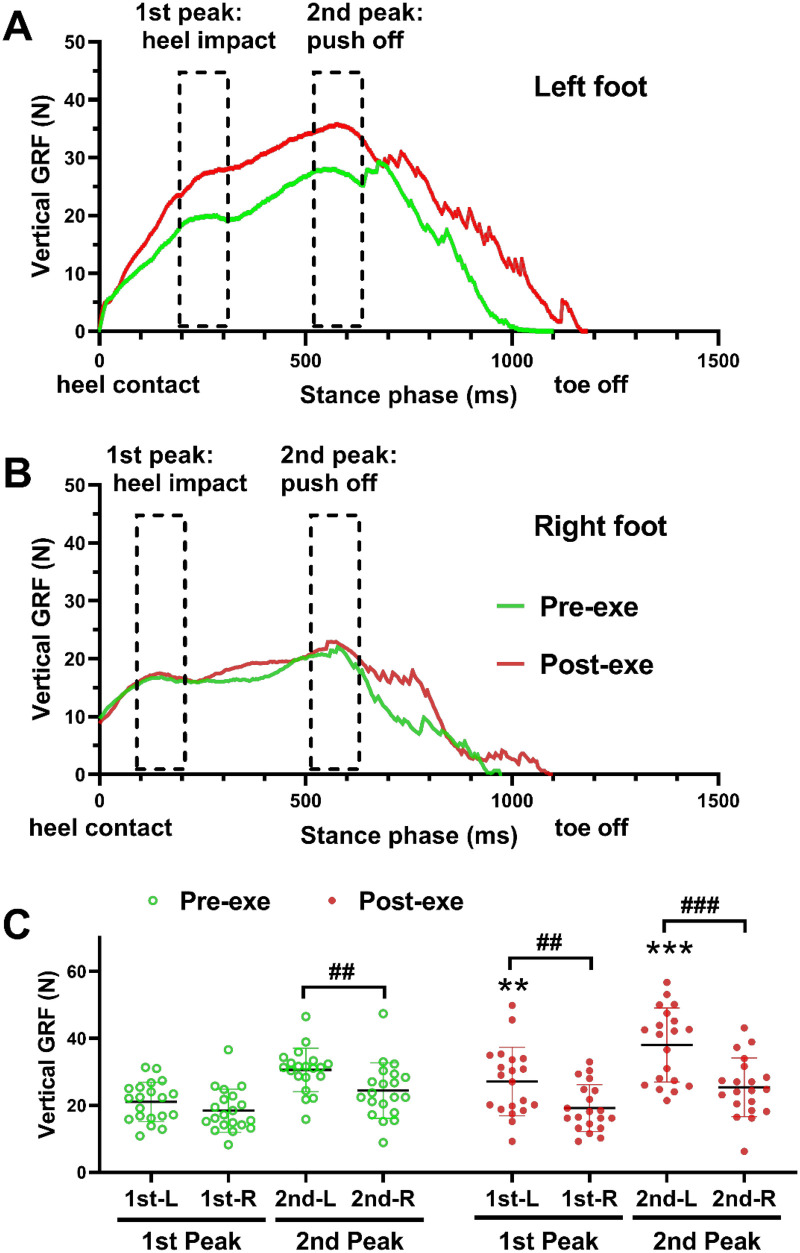
VGRF dynamics before and after the complete CPET and VERT protocol. **(A,B)** Average VGRF profiles across stance and pre-swing phases pre- and post-exercise for the left and right feet, respectively. **(C)** VGRF peak values during heel strike (1st peak) and forefoot push-off (2nd peak) before and after exercise. Data are presented as mean ± SD (*n* = 20). **P < 0.01, ***P < 0.001 vs. pre-exercise values; ^#^*P* < 0.05, ^##^*P* < 0.01, ^###^*P* < 0.001 for comparisons between left and right feet (two-way repeated-measures ANOVA with Bonferroni *post hoc* test). CPET, cardiopulmonary exercise test; VERT, verification test; VGRF, vertical ground reaction force.

VGRF and its passive (heel strike) and active (forefoot push-off) peaks provide critical insight into the kinematic and kinetic adaptations of the lower limb during gait. In the present study, dynamic foot-scan analysis revealed pronounced alterations in plantar loading patterns following completion of the CPET and VERT protocols. Specifically, VGRF significantly increased in the left foot during both the heel strike and forefoot push-off phases, whereas minimal changes were observed in the right foot (Figures 5A,B).

At baseline (pre-exercise), participants consistently exhibited higher VGRF values in the left foot compared with the right. During the forefoot push-off phase, the second VGRF peak was significantly greater in the left foot (31.58 ± 7.76 N) than in the right (24.41 ± 8.05 N; *P* < 0.01; Figure 5C). Following the complete exercise protocol, left-foot VGRF increased substantially during both heel strike (first peak: pre-exercise, 21.55 ± 6.25 N; post-exercise, 28.13 ± 11.24 N; *P* < 0.01, *d* = 0.75) and forefoot push-off (second peak: pre-exercise, 31.58 ± 7.76 N; post-exercise, 39.03 ± 12.01 N; *P* < 0.001, *d* = 0.75; Figure 5C). By contrast, the right foot exhibited only modest, non-significant increases in VGRF during heel strike (first peak: pre-exercise, 18.45 ± 6.29 N; post-exercise, 19.22 ± 6.82 N) and forefoot push-off (second peak: pre-exercise, 24.41 ± 8.05 N; post-exercise, 25.42 ± 8.52 N; Figure 5C). Notably, post-exercise comparisons between limbs confirmed significantly greater VGRF in the left foot at both heel strike (28.13 ± 11.24 N vs. 19.22 ± 6.82 N; *P* < 0.01) and forefoot push-off (39.03 ± 12.01 N vs. 25.42 ± 8.52 N; *P* < 0.001; Figure 5C).

These asymmetrical kinetic adaptations suggest a lateralized fatigue response, with the left lower limb exhibiting greater load redistribution and altered force output following high-intensity exercise. The enhanced left-foot VGRF coincides with previously observed increases in plantar contact area and pressure, indicating that post-fatigue neuromuscular adjustments during stance and propulsion phases may have occurred more prominently on the left side. Importantly, limb dominance was not formally assessed in the present study; therefore, these asymmetries should be interpreted as side-specific adaptations rather than dominance-related compensatory mechanisms. The observed inter-limb differences may reflect individual motor strategies or subtle neuromuscular variability emerging under fatigue conditions. Such lateralized loading patterns could potentially increase localized mechanical stress during prolonged high-intensity locomotion; however, causal inferences regarding preferential loading or injury risk require further investigation.

### 3.6 FPA adaptations

**Figure 6:**
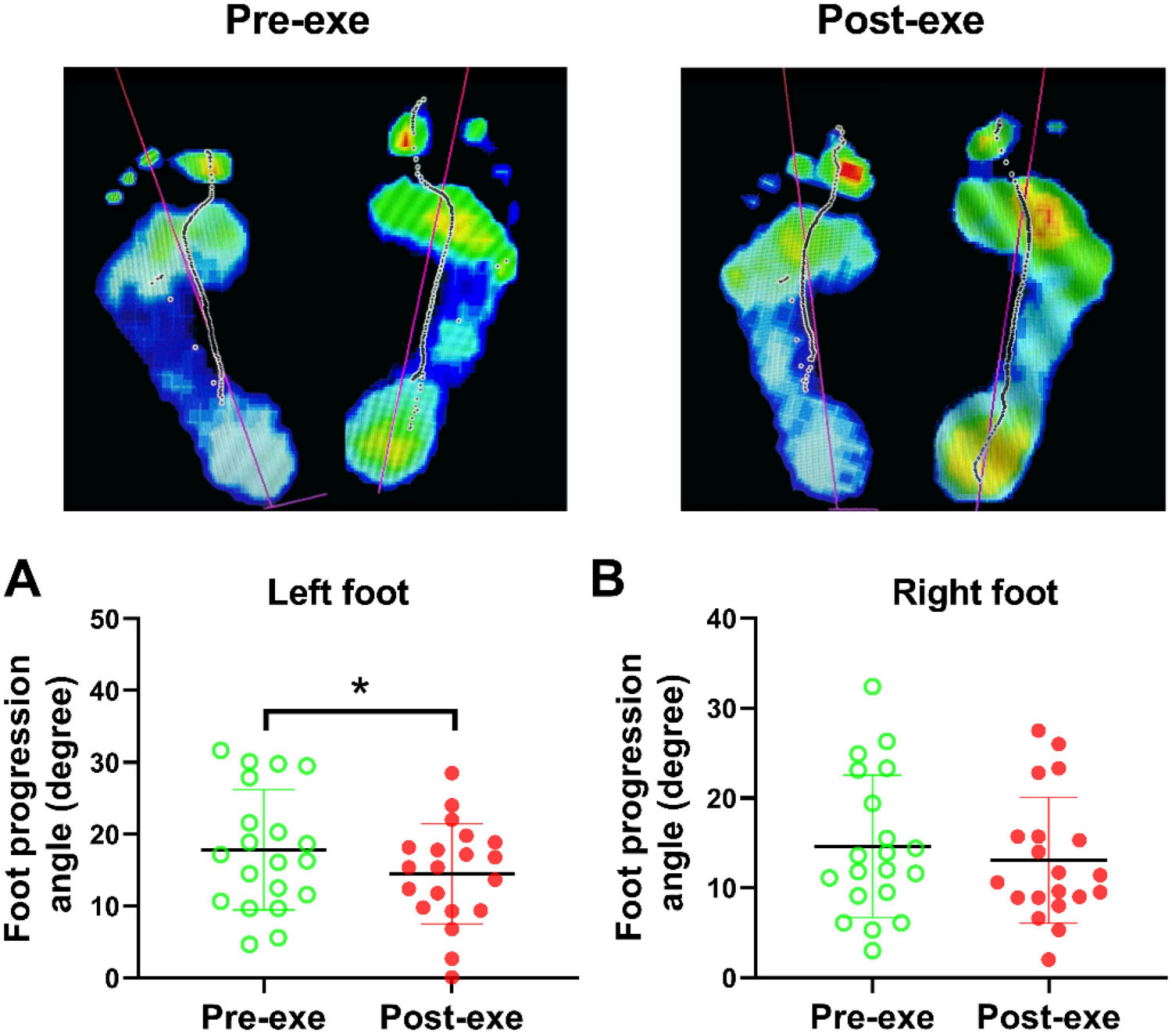
FPA alterations before and after completion of the CPET and VERT protocols. The representative screenshot depicts the change in the FPA, defined as the angle between the longitudinal axis of the foot and the line of progression (indicated by the red line), before and after high-intensity treadmill exercise. **(A,B)**: FPA values for the left and right feet, respectively, pre- and post-exercise. Data are presented as mean ± SD (*n* = 20). **P < 0.01 by paired *t*-test. CPET, cardiopulmonary exercise test; FPA, foot progression angle; VERT, verification test.

Consistent with the aforementioned plantar kinetic alterations, notable changes in the FPA were primarily observed in the left foot following the completion of the CPET and VERT protocols. The mean FPA of the left foot significantly decreased from 17.86 ± 8.17° at baseline to 14.50 ± 6.79° post-exercise (*P* < 0.01, *d* = 0.45; Figure 6A), indicating a shift toward greater internal rotation during the stance phase. This reduction suggests a fatigue-induced modification in gait mechanics, likely reflecting compensatory neuromuscular adjustments to maintain propulsion efficiency under high-intensity loading conditions.

A similar, though non-significant, decreasing trend was detected in the right foot (from 14.62 ± 7.72° to 13.09 ± 6.83°; Figure 6B). The overall pattern of decreased FPA bilaterally, more pronounced on the left side, further supports the presence of asymmetric fatigue-related adaptations in gait control. These findings correspond with the observed increases in left-foot VGRF and plantar pressure distribution, collectively suggesting a lateralized adjustment in locomotor mechanics under fatigued conditions. As limb dominance was not evaluated, these adaptations are described as inter-limb asymmetries rather than dominance-driven effects. Changes in foot progression angle may alter medial–lateral loading distribution during stance; however, whether such modifications represent compensatory stabilization strategies or transient fatigue-induced variability remains to be clarified in future studies.

## 4 Discussion

This study employed a multimodal experimental framework—integrating incremental cardiopulmonary testing, a supramaximal verification phase, sEMG, and pre-/post-exercise plantar kinetics—to comprehensively characterize the physiological and mechanical manifestations of fatigue during exhaustive treadmill running in collegiate athletes. The main findings were that (1) most participants met established physiological criteria for maximal exertion; (2) sEMG-derived activation indices declined from the incremental to the verification phase, indicating neuromuscular fatigue; (3) plantar kinetics revealed region-specific and asymmetric load redistribution, particularly increased midfoot and lateral forefoot loading in the left foot after exhaustive exercise; and (4) although nominal associations were observed between aerobic–metabolic capacity, neuromuscular activity, and plantar loading, these relationships did not remain statistically significant after BH-FDR correction and should therefore be interpreted as exploratory indications of potential multi-system coupling during fatigue progression.

### 4.1 Cardiopulmonary responses and effort verification

While oxygen uptake dynamics remained stable across test phases, confirming high performance consistency, the presence or absence of a clear plateau in oxygen kinetics was not the focus of interpretation. Consistent with recent reviews (1–4), such plateaus are influenced by methodological and analytical factors, including ramp rate, averaging interval, and data processing, and should not be considered in isolation as markers of maximal effort. Instead, employing a supramaximal verification phase provided a robust framework for confirming performance reproducibility while minimizing interpretive bias.

This approach aligns with emerging consensus that performance verification should encompass multiple indicators (metabolic stabilization, ventilatory responses, and neuromuscular effort) rather than a single criterion (3, 17, 20). The reproducibility of key cardiopulmonary metrics across phases thus supports the reliability of the protocol and indicates that subsequent neuromuscular and mechanical changes primarily reflect fatigue adaptation rather than incomplete effort.

### 4.2 Neuromuscular activation and fatigue dynamics

The sEMG results demonstrated clear reductions in both iEMG and RMS amplitudes from the incremental to the verification phase, consistent with progressive declines in motor-unit recruitment and firing synchrony as fatigue accumulated. Parallel decreases in median frequency within the tibialis anterior and rectus femoris suggested reduced conduction velocity and a recruitment shift toward slower, oxidative fibers, characteristic of type I motor-unit predominance under fatigue conditions (6–9).

These findings align with established electromyographic fatigue signatures, wherein amplitude-based parameters (e.g., iEMG, RMS) reflect global activation effort, whereas spectral-domain metrics (e.g., median frequency) indicate peripheral conduction and fiber-type modulation (10, 29). Although moderate associations were observed between cardiopulmonary efficiency and activation amplitudes, these correlations did not survive FDR correction. Therefore, the findings should be interpreted cautiously as preliminary trends rather than definitive evidence that athletes with superior metabolic profiles sustain greater neural drive or delay fatigue onset. Additionally, because sEMG signals were not normalized to maximal voluntary contraction or dynamic reference values, interpretation of absolute activation magnitudes and cross-study comparability may be limited, despite the within-session repeated-measures design minimizing electrode-related variability. Taken together, these observations tentatively support the concept that endurance performance may emerge from coordinated cardiovascular–neuromuscular interactions (7, 30, 31); however, confirmation requires studies with larger samples and pre-specified primary outcomes.

### 4.3 Plantar kinetic adaptations and asymmetry

The Pre–Post comparison revealed significant increases in midfoot contact area (+12.3%), mean pressure (+10.8%), and left-foot peak vertical ground reaction force, confirming that the CPET–VERT sequence elicited measurable post-exercise mechanical adaptations during walking, reflecting residual neuromechanical fatigue rather than in-run kinetic alterations. In both feet, midfoot regions exhibited higher post-exercise loading, with pronounced increases in the left midfoot and lateral forefoot, reflecting compensatory strategies to maintain propulsion during reduced muscular efficiency. These outcomes mirror previous findings that prolonged or high-intensity running induces midfoot engagement and forefoot load shifts as fatigue develops (11, 12, 32).

In particular, the increase in left-foot regional VGRF may reflect altered shock attenuation capacity and side-specific redistribution of vertical loading during stance. Under fatigued conditions, reduced distal muscle efficiency, especially in ankle stabilizers, may necessitate compensatory adjustments in foot-ground interaction to preserve forward progression. The elevated regional VGRF observed on the left side may therefore represent a task-dependent redistribution of mechanical demand rather than a simple imbalance.

The detected asymmetry, characterized by increased midfoot pressure and reduced foot progression angle on the left side, indicates lateralized fatigue responses, consistent with prior reports that fatigue enhances inter-limb asymmetry in plantar pressure and gait kinematics (33, 34). Limb dominance was not formally assessed in the present study; therefore, these asymmetries are described as side-specific or lateralized fatigue responses rather than dominance-driven compensation. Importantly, baseline bilateral symmetry was not quantitatively evaluated prior to the fatigue protocol. Consequently, pre-existing inter-limb variability cannot be excluded, and the observed left-sided loading differences should be interpreted as post-exercise adaptations potentially superimposed upon individual biomechanical characteristics. The observed inter-limb differences may reflect individual motor strategies, transient neuromuscular variability, or task-specific adaptation under fatigued conditions (14, 16). Alterations in medial loading distribution and foot progression angle may modify local mechanical demand; however, causal links to overuse injury risk cannot be inferred from the present cross-sectional design and require longitudinal investigation.

Although negative correlations between aerobic–metabolic efficiency and midfoot pressure were observed, these associations did not remain statistically significant after false discovery rate correction. Given the moderate effect sizes and limited sample size (*n* = 20), statistical power was reduced following adjustment for multiple comparisons. Therefore, these relationships should be considered exploratory rather than confirmatory evidence of enhanced gait stability in individuals with superior physiological economy. Future studies incorporating baseline symmetry quantification and longitudinal fatigue tracking will be necessary to determine whether side-specific VGRF amplification represents a consistent fatigue marker or reflects inter-individual variability. This coupling between energy supply, neural drive, and mechanical stability underscores the multi-system coordination underlying fatigue resistance (30, 31). It should be noted that the VGRF values reported represent regional peak vertical forces detected by the plantar pressure system rather than whole-body ground reaction forces measured via force platforms, which explains their comparatively lower magnitude.

### 4.4 Integrated interpretation and practical implications

Together, the neuromuscular and plantar kinetic findings suggest that fatigue during exhaustive exercise may involve multi-domain adaptations spanning metabolic, neural, and mechanical systems. Importantly, the Pre–Post plantar kinetic results revealed that even in the absence of significant metabolic deterioration, local mechanical adaptations—such as increased midfoot engagement and asymmetrical loading—serve as sensitive indicators of neuromechanical fatigue. This underscores that gas-exchange parameters alone cannot fully capture the functional consequences of fatigue on movement efficiency.

From an applied perspective, integrating neuromuscular (sEMG) and plantar kinetic assessments with standard cardiopulmonary testing offers a more comprehensive method for fatigue monitoring and performance evaluation. Detecting disproportionate midfoot loading or lateral asymmetry following high-intensity exercise may provide preliminary indicators of neuromechanical fatigue; however, further validation is required before clinical or injury-prevention applications can be established. Importantly, the present findings should not be interpreted as evidence of pathological asymmetry but rather as potential adaptive responses under acute fatigue conditions. Such multimodal insights can aid in refining recovery strategies, adjusting training loads, and preventing overuse injuries in athletes. The present findings highlight that maintaining coordination between metabolic and mechanical efficiency is critical for sustaining performance as physiological limits are approached.

### 4.5 Limitations and future directions

The present findings should be interpreted in light of several limitations. First, the sample consisted of 20 trained male athletes, which restricts generalizability across sex, age, and training status. The relatively small sample size may also have limited statistical power, particularly for correlational analyses. As a result, several moderate associations did not retain statistical significance after false discovery rate correction and should therefore be considered exploratory. Second, sEMG signals were not normalized to maximal voluntary contraction or dynamic reference values. Although the within-session repeated-measures design minimized electrode-related variability, the absence of normalization may limit cross-study comparability and interpretation of absolute activation magnitudes. Third, plantar kinetic assessments were conducted during walking rather than running. While this approach enabled standardized and reliable regional pressure analysis, the findings reflect residual post-exercise gait adaptations rather than direct in-run biomechanics. Caution is therefore warranted when extrapolating these results to running-phase mechanics.

Additionally, biochemical indices such as blood lactate concentration or muscle oxygenation were not assessed. Integrating metabolic, neuromuscular, and biomechanical markers within the same experimental framework would provide a more comprehensive characterization of fatigue mechanisms. Future research should employ larger and more diverse cohorts, incorporate standardized EMG normalization procedures, and integrate high-density EMG, wearable sensing technologies, or advanced analytical approaches (e.g., machine-learning modeling) to characterize individualized fatigue signatures across different exercise modalities.

## 5 Conclusion

This study demonstrates that fatigue during exhaustive treadmill running emerges from tightly integrated cardiopulmonary, neuromuscular, and biomechanical adaptations. sEMG revealed reduced activation amplitude and conduction frequency, indicating diminished motor-unit recruitment and synchronization, while pre–post plantar kinetics showed asymmetric midfoot loading and compensatory redistribution, particularly in the left foot. These findings suggest that fatigue extends beyond metabolic stabilization, involving coordinated neuromechanical adjustments; however, the inter-system relationships observed should be interpreted cautiously pending confirmation in larger-scale studies. The multimodal framework combining CPET, sEMG, and plantar analysis offers a comprehensive means to detect fatigue-related transitions, enhance performance evaluation, and inform injury-prevention strategies in athletic populations.

## Data Availability

The original contributions presented in the study are included in the article/Supplementary Material, further inquiries can be directed to the corresponding author.
