# Supplementary material for: Multimodal assessment of exercise-induced fatigue: integrating cardiopulmonary, neuromuscular, and biomechanical profiling in high-intensity running
Source: Front Sports Act Living. 2026 Mar 25;8:1779542. doi: 10.3389/fspor.2026.1779542 (PMC13057562; doi:10.3389/fspor.2026.1779542)
Supplement: Supplementary file 1 [file Table1.docx]

**ONLINE RESOURCES (supplementary materials)**

**Multimodal Assessment of Exercise-Induced Fatigue: Integrating Cardiopulmonary, Neuromuscular, and Biomechanical Profiling in High-Intensity Running**

- **Running title: Integrated Assessment of Exercise Fatigue in High-Intensity Running**

Yekai Wang^#^, Ang Chen^#^, Yichen Xu, Xindong Tao, Jihui Wang, Hui Li, Wei Ouyang*

College of Physical Education and Health Sciences, Zhejiang Normal University, Jinhua 321004, China

^#^These authors contributed equally to this work.

*** Correspondence:**Wei Ouyang, PhD

College of Physical Education and Health Sciences

Zhejiang Normal University

288 Yingbin Road

Jinhua 321004, China

Email: wouyang@zjnu.cn

Phone: 86-0579-8228257

**Supplementary Table S1 The participants’ basic data**

| **Participant** | **Age**  **(yr)** | **Heght (cm)** | **Weight (kg)** | **BHR (BPM)** | **SBP (mmHg)** | **DBP (mmHg)** | **Overall fat %** | **BMI**  **(kg/m^2^)** | **BMR (KJ)** | |  |
| --- | --- | --- | --- | --- | --- | --- | --- | --- | --- | --- | --- |
| #1 | 20 | 175 | 67 | 64 | 122 | 65 | 6.9 | 20.7 | 7335 | |  |
| #2 | 23 | 175 | 66.5 | 100 | 122 | 85 | 8.8 | 21.7 | 7213 | |  |
| #3 | 24 | 173 | 75 | 76 | 108 | 68 | 19.6 | 25.1 | 7594 | |  |
| #4 | 21 | 175 | 67 | 69 | 111 | 62 | 12.3 | 21.8 | 7468 | |  |
| #5 | 19 | 182 | 62 | 56 | 128 | 74 | 6.8 | 18.7 | 7082 | |  |
| #6 | 24 | 176 | 68 | 67 | 110 | 71 | 15.8 | 21.9 | 7783 | |  |
| #7 | 21 | 178 | 62 | 73 | 114 | 66 | 8.8 | 19.6 | 7636 | |  |
| #8 | 23 | 170 | 60 | 58 | 124 | 67 | 9.7 | 20.7 | 7481 | |  |
| #9 | 23 | 174 | 74 | 87 | 117 | 68 | 13.2 | 24.4 | 7719 | |  |
| #10 | 21 | 180 | 70 | 65 | 117 | 63 | 10.9 | 21.3 | 7653 | |  |
| #11 | 20 | 180 | 60 | 58 | 106 | 55 | 6.7 | 18.5 | 7678 | |  |
| #12 | 24 | 181 | 84 | 77 | 107 | 69 | 19.4 | 25.7 | 8167 | |  |
| #13 | 23 | 172 | 70 | 75 | 115 | 68 | 17.4 | 23.6 | 7884 | |  |
| #14 | 19 | 170 | 64.5 | 64 | 129 | 51 | 8.9 | 22.3 | 7142 | |  |
| #15 | 21 | 183 | 70 | 62 | 109 | 62 | 18.8 | 21.0 | 6937 | |  |
| #16 | 23 | 170 | 76.5 | 66 | 121 | 73 | 18.9 | 26.5 | 7498 | |  |
| #17 | 24 | 182 | 70 | 84 | 122 | 70 | 8.3 | 21.1 | 7611 | |  |
| #18 | 21 | 178 | 62 | 60 | 117 | 64 | 8.0 | 19.5 | 6841 | |  |
| #19 | 23 | 177 | 82.5 | 89 | 123 | 75 | 18.3 | 26.3 | 8238 | |  |
| #20 | 21 | 175 | 68 | 62 | 127 | 51 | 7.2 | 22.2 | | 7684 | |

n=20. Abbreviations: BHR, basal heart rate; BMI, body mass index; BMR, basal metabolic rate; BPM, beat per minute; DBP,

diastolic blood pressure; SBP, systolic blood pressure.

**Supplementary Table S2 The participants’ fitness data (n=20)**

| Test Participant  Variables | #1 | #2 | #3 | #4 | #5 | #6 | #7 | #8 | #9 | #10 | #11 | #12 | #13 | #14 | #15 | #16 | #17 | #18 | #19 | #20 |
| --- | --- | --- | --- | --- | --- | --- | --- | --- | --- | --- | --- | --- | --- | --- | --- | --- | --- | --- | --- | --- |
| Single-leg balance (Max 60 seconds) | 60 | 60 | 60 | 60 | 60 | 60 | 60 | 60 | 60 | 60 | 60 | 60 | 60 | 60 | 60 | 60 | 60 | 60 | 60 | 60 |
| Number of sit-to-stand in 30 seconds (arms crossed in chest) | 33 | 19 | 30 | 33 | 31 | 27 | 38 | 33 | 30 | 35 | 38 | 18 | 30 | 30 | 29 | 17 | 30 | 32 | 30 | 35 |
| Right arm curl count with a 5 kg dumbbell (30 seconds) | 28 | 19 | 30 | 24 | 26 | 32 | 30 | 28 | 26 | 26 | 32 | 22 | 27 | 34 | 28 | 24 | 28 | 25 | 32 | 32 |
| Distance from extended finger to toes in a seated position | 26 | -4.5 | 14 | 28 | 12 | 8 | 8.5 | 18 | 12 | 20 | 3.5 | 3.5 | 9 | 4 | 19 | 15 | 8 | 23 | 10 | 19 |
| Back scratch test distance (distance between middle fingers) | 5 | 3 | 7 | 6 | 7 | 2 | 5 | 5 | 8 | 6 | 2 | 6 | 3 | 4 | 2 | -3.5 | 8 | 5 | 2 | 8 |
| Time to walk 2.45 meters, turn, and return to seat | 6.8 | 8.6 | 7.45 | 7 | 7.03 | 7.68 | 6.43 | 6.53 | 8.08 | 6.3 | 6.8 | 6.27 | 7.86 | 6.59 | 7.2 | 8.49 | 6.83 | 6.5 | 7.15 | 6.62 |
| 30m walking speed | 1.48 | 1.39 | 1.42 | 1.22 | 1.58 | 1.61 | 1.78 | 1.41 | 1.38 | 1.41 | 1.60 | 1.37 | 1.46 | 1.44 | 1.35 | 1.43 | 1.56 | 1.48 | 1.50 | 1.32 |
| 6-minute walking speed | 1.21 | 1.20 | 1.18 | 1.12 | 1.16 | 1.25 | 1.36 | 1.15 | 1.10 | 1.18 | 1.25 | 1.10 | 1.21 | 1.15 | 1.15 | 1.19 | 1.24 | 1.20 | 1.20 | 1.08 |

**Supplementary Table S3 The participants’ blood pressure and heart rate changes**

|  | BP at Pre-exe | | BP at Recovery | | BP at 10 min post-exe | | Heart rate changes (BPM) | | | | | |
| --- | --- | --- | --- | --- | --- | --- | --- | --- | --- | --- | --- | --- |
| Participants | SBP  (mmHg) | DBP  (mmHg) | SBP (mmHg) | DBP (mmHg) | SBP (mmHg) | DBP  (mmHg) | Pre-exe HR | CPET HR | Recovery-HR | VERT-HR | 2 min post-exe HR | 10 min post-exe HR |
| #1 | 122 | 65 | 144 | 71 | 141 | 73 | 64 | 199 | 118 | 193 | 174 | 124 |
| #2 | 122 | 85 | 125 | 82 | 133 | 83 | 100 | 199 | 144 | 200 | 190 | 133 |
| #3 | 108 | 68 | 125 | 75 | 124 | 77 | 76 | 189 | 124 | 190 | 155 | 128 |
| #4 | 111 | 62 | 124 | 64 | 123 | 58 | 69 | 186 | 117 | 187 | 146 | 118 |
| #5 | 128 | 74 | 123 | 80 | 125 | 76 | 56 | 187 | 100 | 190 | 165 | 106 |
| #6 | 110 | 71 | 116 | 69 | 113 | 67 | 67 | 197 | 113 | 192 | 170 | 111 |
| #7 | 114 | 66 | 143 | 81 | 102 | 71 | 73 | 216 | 154 | 213 | 190 | 137 |
| #8 | 124 | 67 | 134 | 76 | 114 | 76 | 58 | 184 | 102 | 196 | 156 | 102 |
| #9 | 117 | 68 | 130 | 61 | 132 | 63 | 87 | 201 | 132 | 203 | 170 | 132 |
| #10 | 117 | 63 | 122 | 62 | 106 | 71 | 65 | 198 | 121 | 189 | 153 | 123 |
| #11 | 106 | 55 | 111 | 58 | 123 | 64 | 58 | 184 | 110 | 181 | 146 | 112 |
| #12 | 107 | 69 | 122 | 63 | 136 | 75 | 77 | 192 | 110 | 189 | 147 | 117 |
| #13 | 115 | 68 | 131 | 80 | 126 | 77 | 75 | 196 | 128 | 185 | 160 | 120 |
| #14 | 129 | 51 | 136 | 72 | 128 | 68 | 64 | 186 | 124 | 193 | 152 | 128 |
| #15 | 109 | 62 | 129 | 65 | 125 | 61 | 62 | 194 | 105 | 186 | 152 | 110 |
| #16 | 121 | 73 | 115 | 67 | 122 | 75 | 66 | 203 | 116 | 199 | 170 | 114 |
| #17 | 122 | 70 | 144 | 78 | 142 | 82 | 84 | 195 | 140 | 197 | 159 | 141 |
| #18 | 117 | 64 | 116 | 56 | 130 | 64 | 60 | 182 | 109 | 178 | 150 | 110 |
| #19 | 123 | 75 | 119 | 70 | 137 | 70 | 89 | 202 | 110 | 200 | 149 | 109 |
| #20 | 127 | 51 | 109 | 55 | 117 | 64 | 62 | 194 | 112 | 194 | 160 | 105 |

n=20. Abbreviations: BMI, body mass index; BMR, basal metabolic rate; BP, blood pressure; BPM, beat per minute; CPET, cardiopulmonary exercise testing; DBP, diastolic blood pressure; HR, heart rate; SBP, systolic blood pressure.

**Supplementary Table S4 The participants’ rating of perceived exertion after incremental CPET and VERT**

| **Grade** | **Rating of Perceived Exertion** | **RPE(n)-CPET** | **RPE(n)-VERT** |
| --- | --- | --- | --- |
|  |  |  |  |
| 6 | No exertion at all |  |  |
| 7 | Extremely light |  |  |
| 8 |  |  |  |
| 9 | Very light |  |  |
| 10 |  |  |  |
| 11 | Light |  |  |
| 12 | Somewhat hard |  |  |
| 13 |  |  |  |
| 14 |  |  |  |
| 15 | Hard(heavy) |  |  |
| 16 | Very hard |  |  |
| 17 |  |  |  |
| 18 |  | 6 | 3 |
| 19 | Extremely hard | 9 | 14 |
| 20 | Maximal exertion | 5 | 3 |

n=20. Abbreviations: CPET, cardiopulmonary exercise testing; VERT, verification testing.

**Supplementary Table S5 Pearson correlation analysis and Benjamini–Hochberg false discovery rate (FDR) correction (Q = 0.05)**

| Comparison | r value | Raw P value | BH-adjusted P value (Q=0.05) | Significant (Raw P < 0.05) | Significant after BH-FDR (Q=0.05) |
| --- | --- | --- | --- | --- | --- |
| VO2max-CPET vs VO2max-VERT | 0.73 | 0.0003 | 0.0021 | Yes | Yes |
| VO2max vs LTA iEMG (CPET) | 0.47 | 0.037 | 0.056 | Yes | No |
| VO2max vs RBF iEMG (VERT) | 0.43 | 0.058 | 0.058 | No | No |
| VO2max vs RBF RMS (VERT) | 0.468 | 0.038 | 0.056 | Yes | No |
| VO2max vs LTA MF (VERT) | -0.447 | 0.048 | 0.056 | Yes | No |
| VO2max vs Right Midfoot Contact Area (Post) | -0.47 | 0.047 | 0.056 | Yes | No |
| VO2max vs Right Midfoot Pressure (Post) | -0.477 | 0.034 | 0.056 | Yes | No |

Abbreviations: CPET, cardiopulmonary exercise testing; iEMG, integrated EMG; LTA, left tibialis anterior; MF, Median Frequency; RBF, right biceps femoris; RMS, root mean square; VERT, verification testing.
